# Psychotropic drug use among 0–17 year olds during 2004–2014: a nationwide prescription database study

**DOI:** 10.1186/s12888-016-0716-x

**Published:** 2016-01-29

**Authors:** Ingeborg Hartz, Svetlana Skurtveit, Anne Kjersti Myhrene Steffenak, Øystein Karlstad, Marte Handal

**Affiliations:** Faculty of Public Health, Hedmark University College, Elverum, Norway; Division of Epidemiology, Norwegian Institute of Public Health, Oslo, Norway; Norwegian Centre for Addiction Research, University of Oslo, Oslo, Norway

**Keywords:** Time trends, Drug utilization, Psychotropic drugs, Children, Adolescents, Prescription database, Norway

## Abstract

**Background:**

Time-trend studies on psychotropic drugs among children and adolescents are scarce, and most of them are outdated. The purpose of this study was to study prevalences of psychotropic drug use during 2004–2014 among Norwegians aged <18 years, overall and in psychotropic sub-groups.

**Methods:**

Data were obtained from the Norwegian Prescription Database, which covers all dispensed prescription drugs in Norway from 2004 and onwards. Psychotropic drugs included: antipsychotics (ATC-group N05A), anxiolytics (N05B), hypnotic/sedatives (N05C), antidepressants (N06A), stimulants (N06BA), and alimemazine (R06AD01). Period (1-year) prevalence of use, overall and in subgroups of psychotropic drugs, was estimated by identifying individuals <18 years who had at least one psychotropic drug dispensed during each year.

**Results:**

Psychotropic drug use increased in 0–17 year olds over an 11-year period, in which the main contributing drugs were stimulants (boys overall; 15.0 to 20.8/1000, girls overall; 3.8 to 8.5/1000), hypnotic/sedative drugs in adolescents (boys overall; 4.2 to 10.8/1000, girls overall; 2.6 to 8.8/1000) and to some extent antidepressants among adolescent girls (girls overall from 3.1 to 4.0/1000). Psychotropic drug use was, however, reduced by half in the youngest children, attributed to reduction of alimemazine only (1-year olds: boys; from 36.6 to 10.2/1000, girls; 26.9 to 7.2/1000). A higher level of psychotropic drug use was observed among younger boys, but there is a shift towards girls using more psychotropic drugs than boys during adolescence for all psychotropic drugs except for stimulants.

**Conclusion:**

Different trends in psychotropic drug use exist in age and gender subgroups. Psychotropic drug use has decreased among the youngest children, attributed to alimemazine, and increased in older children and adolescents, attributed mainly to stimulants and hypnotics/sedatives.

## Background

Time-trend studies on psychotropic drugs among children and adolescents are scarce, and most of them are outdated. Overall, there seem to be similar trends of increasing use of psychotropic drugs in children and adolescents in the recent past in European countries [1], Netherlands (1995–2000) [2], Denmark (1996–2010) [3], Iceland (2003–2007) [4] and Norway (2004–2010 in 15–16 year olds) [5]. In parallel, psychotropic drug use in the US among children and adolescents increased twofold to threefold from 1984 to 1996, and increased further between 2000 and 2002 [1, 6].

The increase has been attributed to use of stimulants in particular, as observed in European countries [2–4, 7, 8]. However, although there has been a trend of increasing use of stimulants, the level of use varies substantially between countries. For example, in a Nordic comparison study, Zoega et al. documented that 7–15 year old Icelanders were nearly five times more likely than Finnish children at the same age to have used ADHD drugs in 2007 [9].

Time trends for use of antidepressants vary between countries. For example, whereas an increasing use of antidepressants among 0–17 year olds is observed in Denmark between 1995 and 2011 [3, 10], use in this age-group decreased in Iceland in the period 2003–2007 [4]. A trend of increasing use of antidepressants was observed in Canada between 2005 and 2009 [11].

Antipsychotic drug use among 0–17 year olds has also been increasing in Iceland (up to 2007) [4], and Denmark (up to 2010) [3], but at a higher level in Iceland. An increase has also been observed in Canada up to 2010 [12, 13].

Time-trend studies on the use of hypnotic drugs in children and adolescents are scarce, since many studies of psychotropic drugs in children and adolescents exclude hypnotic drugs. The few published studies so far reveal a trend of increasing use [4, 14]. For example, overall, hypnotic drug use among 0–17 year old Norwegians increased in the period 2004–2011, due to an increasing use of melatonin in particular [14]. In parallel, use of the sedative antihistamine alimemazine declined in 0–17 year old Norwegians in the period [14]. In Norway alimemazine is licensed for use as a hypnotic drug in children aged 2 years and older, and has long been used for childhood insomnia in Norway [15, 16].

Anxiolytics is one of the psychotropic subgroups prescribed more seldom to children and adolescents, and the few studies published show no large changes in prescribing over time [3, 4].

Considering the lack of up-to-date information on psychotropic drug use, and the focus on off-label use by children and adolescents, new studies on psychotropic drug use by this population are warranted. For example, in 2004–05 European (European Medicines Agency, EMA) and US (Food and Drug Administration, FDA) drug medical agencies issued warnings against the use of antidepressants in youths [17, 18], which emphasize the need for pharmacoepidemiological studies in the post-warning period.

The nationwide Norwegian Prescription Database (NorPD) captures completely dispensed drugs on an individual level as prescribed to patients in ambulatory care [19]. The aims of this study were to examine the prevalence of and trends in psychotropic drug use in the period 2004–2014, overall and in psychotropic drug subgroups in the entire population of Norway aged <18 years.

## Methods

### Data source: the Norwegian prescription database

Prescription data on psychotropic drugs in 2004–2014 were drawn from NorPD which covers the entire nation (5.1 million inhabitants) [19]. From January 2004, all Norwegian pharmacies have been obliged to submit data electronically to the Norwegian Institute of Public Health on all prescribed drugs (irrespective of reimbursement) dispensed to individuals in ambulatory care. The drugs are classified according to the Anatomical Therapeutic Chemical (ATC) classification system [20]. Anonymous data collected for our study were patients’ unique identity number (encrypted), sex, age, the year of dispensing, and drug information (ATC code). All prescription data in Norway is publicly available, with possibilities to create reports on the number of users of a particular drug or drug group. The data can be split by sex, 5-year age groups and geography [19]. The data collected for this study included age on all individuals as a variable, and data access was granted by the Norwegian Institute of Public Health. Use of anonymous register-data does not require any approval from the Regional Committees for Medical and Health Research Ethics, or notification to the Norwegian Data Inspectorate or Data Protection Officer [19].

Our study sample comprised all Norwegian inhabitants below 18 years of age (1.1 million), registered with a valid personal identity number in NorPD. Information was retrieved for the period 2004 to 2013 on the use of all relevant psychotropic drugs: antipsychotics (ATC-group N05A), anxiolytics (N05B), hypnotic/sedative drugs (N05C), antidepressants (N06A), centrally acting sympathomimetics, referred to as stimulants (N05BA), and alimemazine (R06AD01). The systemic antihistamine alimemazine is licensed in Norway for use as a supplement to behavioural therapy in childhood insomnia for children aged 2 years and older, and has been available on the market in Norway since 1975 [21]. It has for a long time been prescribed relatively frequently to young children for sleep problems [15, 16, 22]. It is on this basis that alimemazine was included. All these drug subgroups will be referred to as ‘psychotropic drugs’.

### Analyses and statistics

Period (1-year) prevalence of the use of psychotropic drugs, overall and in subgroups of psychotropic drugs, was estimated by identifying individuals <18 years who had at least one psychotropic drug dispensed during each year. One-year prevalence for psychotropic drugs overall was calculated by identifying individuals who had at least one psychotropic drug dispensed from any of the included psychotropic drug subgroups, and individuals were not counted more than once even if they had multiple psychotropic drugs dispensed during a year. The denominator (inhabitants <18 years) is the total number of inhabitants in Norway per 1^st^ of July in each year, as registered by Statistics Norway (Table 1).

**Table 1:** One-year prevalence (per 1000 inhabitants < 18 years of age) of psychotropic drug use among 0–17 year olds ^a^; overall and in psychotropic drug subcategories, in the period 2004-2014

*Boys*	2004	2005	2006	2007	2008	2009	2010	2011	2012	2013	2014
* N=*	555,198	557,821	560,169	561,730	563,373	565,599	568,891	571,362	573,463	575,654	576,584
Alimemazine (R06DA01)	8.3	7.1	7.0	7.0	5.8	5.7	6.3	5.6	4.9	4.4	4.5
Hypnotics/sedatives (N05C)	4.2	5.3	6.1	7.3	7.9	8.7	9.5	10.0	10.1	10.1	10.8
Stimulants (N06BA)	15.0	16.9	17.5	18.7	19.7	20.5	21.0	20.8	20.7	20.6	20.8
Antidepressants (N06A)	2.1	1.7	1.7	1.8	1.8	2.0	2.2	2.2	2.2	2.1	2.0
Antipsychotics (N05A)	1.6	1.8	1.9	2.0	2.2	2.3	2.3	2.4	2.3	2.2	2.2
Anxiolytics (N05B)	4.7	4.8	4.9	4.9	4.5	4.6	4.6	4.7	4.6	4.2	3.9
Total psychotropics	30.6	30.7	31.8	33.5	33.5	35.0	37.0	36.7	35.9	34.7	35.3
*Girls*	2004	2005	2006	2007	2008	2009	2010	2011	2012	2013	2014
* N=*	527,128	530,212	532,559	534,273	535,906	537,882	540,265	543,012	544,762	547,243	548,577
Alimemazine (R06DA01)	7.1	6.4	6.4	6.4	5.3	4.9	5.5	5.5	5.1	4.6	4.7
Hypnotics/sedatives (N05C)	2.6	3.2	3.7	4.4	5.1	5.7	6.4	7.0	7.7	8.0	8.8
Stimulants (N06BA)	3.8	4.9	5.5	6.3	7.0	7.4	7.9	7.9	8.1	8.3	8.5
Antidepressants (N06A)	3.1	2.5	2.4	2.6	2.6	2.6	2.8	3.0	3.6	4.0	10.0
Antipsychotics (N05A)	1.1	1.1	1.1	1.1	1.1	1.1	1.1	1.2	1.5	1.5	1.6
Anxiolytics (N05B)	4.5	4.7	4.7	4.7	4.4	4.4	4.2	4.4	4.5	4.1	3.9
... Total psychotropics	19.2	19.0	19.7	20.9	20.7	21.1	22.9	23.6	24.5	24.2	25.0

^a^age composition of the overall Norwegian population aged 0–17 years did not change significantly in the period 2004–2014

## Results

### Overall trends in psychotropic drug use among <18 year olds during 2004–2014

**Fig. 1:**
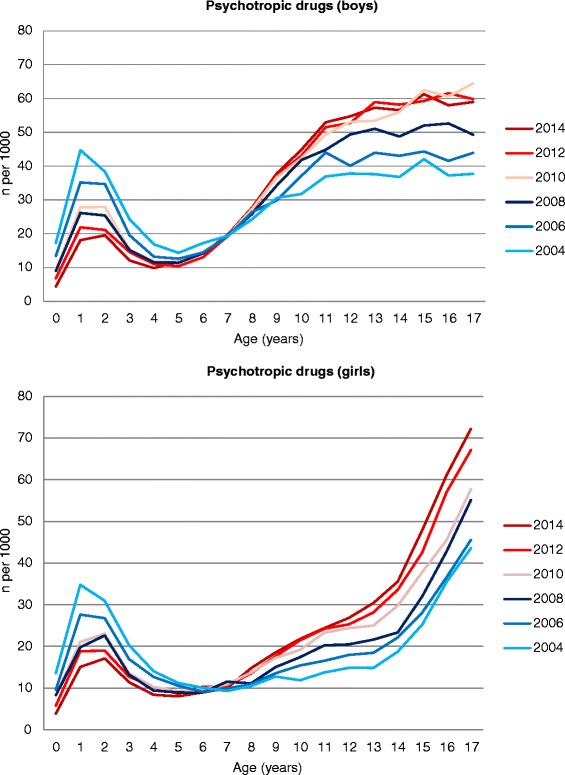
One-year prevalence (per 1000 inhabitants <18 years of age) of psychotropic drug use, by age and gender

Overall use of psychotropic drugs in 0–17 year olds increased slightly in both genders; from 30.6 to 35.3 per thousand inhabitants among boys and from 19.2 to 25.0 per thousand inhabitants among girls (Table 1). The overall level of psychotropic drug use was higher in boys compared to girls in the period, attributed to higher use in male children. However, in later adolescence the level of psychotropic drug use was higher among girls (Fig. 1). In both genders, the main contributor to the overall increase in the period was increasing use of hypnotic/sedatives and stimulants.

**Fig. 2:**
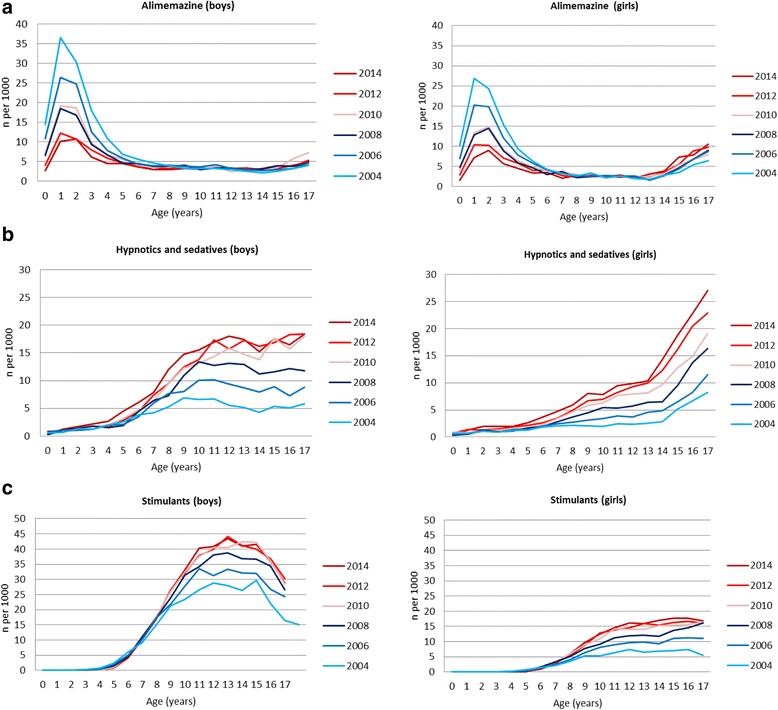
One-year prevalence (per 1000 inhabitants <18 years of age) of use of alimemazine, hypnotics and sedatives, and stimulants in boys and girls, by age

There was a decrease during the study period in overall use of psychotropic drugs in the youngest boys and girls, and among 1–2 year olds in particular (Fig. 1). The decline of psychotropic drug use in 1–2 year olds was attributed to a decreased use of alimemazine, decreasing from 36.6 to 10.2/1000 in 1-year old boys and from 26.9 to 7.2/1000 in girls in the period 2004 to 2014 (Fig. 2a). Thus, the level of alimemazine use in the youngest children declined towards the level of use in older age groups (which remained stable). Use of the traditional hypnotic/sedative drugs among 1–2 year olds remained stable in the period in both genders.

**Fig. 3:**
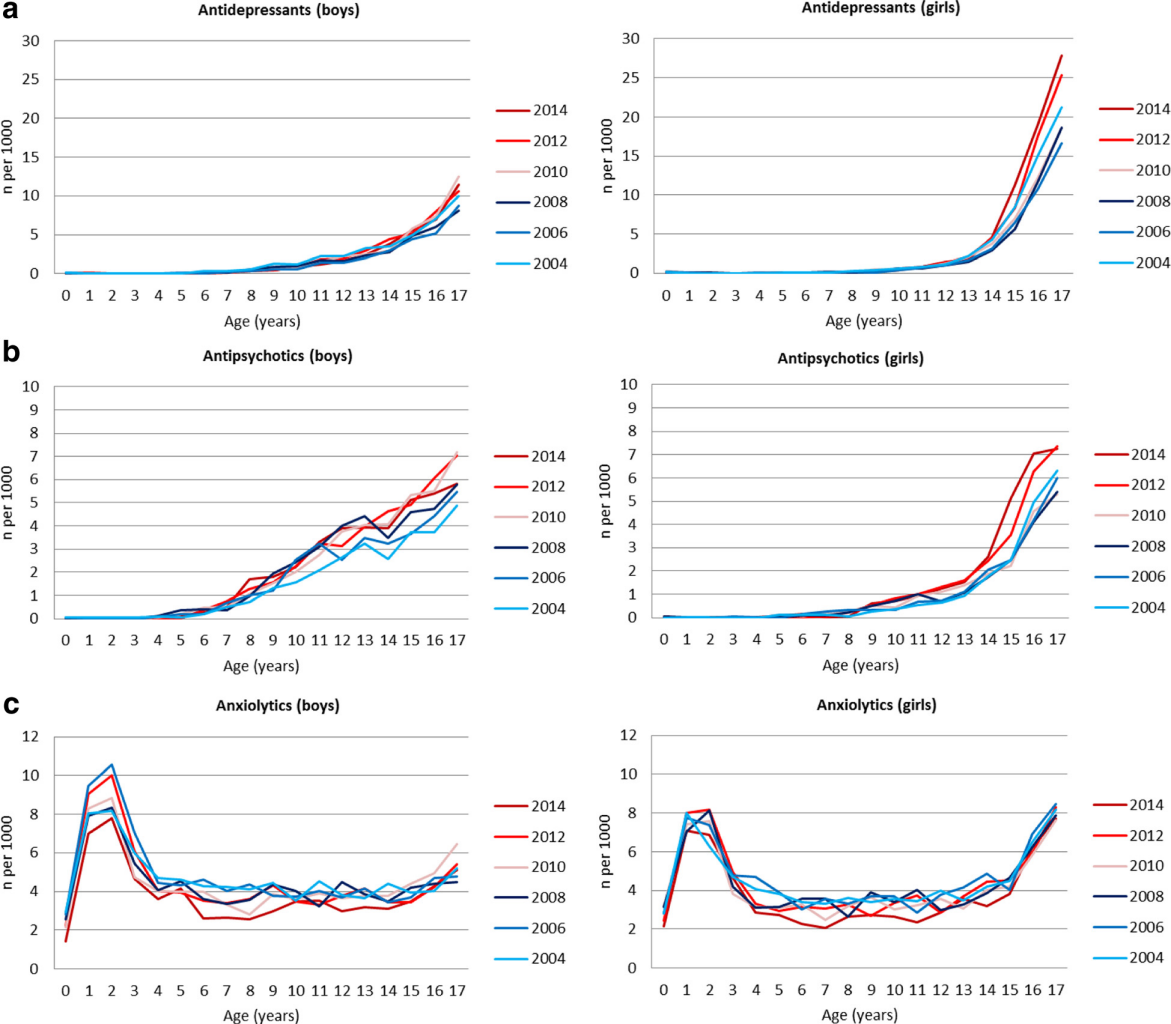
One-year prevalence (per 1000 inhabitants <18 years of age) of use of antidepressants, antipsychotics and anxiolytics in boys and girls, by age

From the age of 8 years upwards, an annual increase in psychotropic drug use was observed in both genders (Fig. 1). This increase in the oldest age groups was attributed to an increasing use of hypnotic and sedative drugs (Fig. 2b), stimulants (Fig. 2c), and to some extent antidepressants (Fig. 3a) as described below.

- *Alimemazine*: alimemazine was the only psychotropic drug whose use decreased in the period in both genders, most pronouncedly in boys (from 8.3 to 4.5/1000 in boys, and 7.1 to 4.7/1000 in girls) (Table 1).
- *Hypnotic and sedative drugs*: use of hypnotic and sedative drugs increased twofold to threefold in both genders in the period (from 4.2 to 10.8/1000 in boys, and from 2.6 to 8.8/1000 in girls) (Table 1). The increase was attributed to an annual increase for the age group 6–17 years in both genders (Fig. 2b). Age trends revealed higher levels of use with older age in both genders. However, whereas use reached a peak at the age of 10–11 in boys, there was an increase in use with age in girls (Fig. 2b).
- *Stimulants*: stimulants increased up to twofold during the period (from 15.0 to 20.8/1000 in boys and 3.8 to 8.5/1000 in girls) (Table 1). However, use of stimulants seems to have been stable in boys and girls from 2010 and onwards. From the age of 6, stimulant use increased with increasing age, reaching a peak of 43.4/1000 in 13-year old boys in 2014 (27.9/1000 in 2004), and then use of stimulants declined steeply towards the age of 17 (Fig. 2c). We also observed an increase in use over time and with increasing age in girls up to 11–12 years of age, then levelling out. The overall relative increases in stimulant use in the period, however, were 120 % among girls and 40 % among boys.
- *Antidepressants:* the time trend in the use of antidepressants revealed an overall decrease in use in boys and girls from 2004 to 2005–6, followed by an annual increase in the following years up to 2014 (Table 1). This increase was more pronounced among girls than boys, attributed to increasing use among those aged 15 and older (Fig. 3a). Antidepressants were used at twice the level among adolescent girls as compared to boys. For example, 27.9/1000 of all 17-year old girls had at least one antidepressant prescription dispensed in 2014, compared to 11.5/1000 in boys.
- *Antipsychotic drugs*: a relative increase in use of about 40 % was observed in the period in both genders (from 1.6 to 2.2/1000 in boys and from 1.1 to 1.65/1000 in girls) (Table 1), attributed to an increase in use from the age of 8 onwards (Fig. 3b). However, the prevalences of use in girls and boys are low.
- *Anxiolytics*: use of anxiolytics was stable in the period (4.7-3.9/1000 in boys and 4.5-3.9/1000 in girls). Throughout the period, overall use of anxiolytics had a peak in 1–2 year olds in both genders (Fig. 3c). Among 1–2 year olds, 97 % of all anxiolytics dispensed were rectal solutions of benzodiazepines (results not shown in figure). Among adolescent girls, use of anxiolytics increased from 15 years of age.

## Discussion

This study revealed an overall increase in psychotropic drug use in 0–17 year olds over a 10-year period, in which the main contributing drugs were stimulants, hypnotic/sedative drugs in adolescents and, to some extent, antidepressants among girls. Psychotropic drug use was, however, reduced by half in the youngest children, attributed to reduction of alimemazine use. A higher level of psychotropic drug use was observed among younger boys, but there is a shift towards girls using more psychotropic drugs than boys during adolescence for all psychotropic drugs except stimulants.

With this study, we present the most comprehensive nationwide analysis to date of psychotropic drug use among children and adolescents over time. Our study has several important strengths. First, it is based on complete data on dispensed psychotropic drugs in out-patient care [23]. Second, all of the drugs included in our analysis are captured by the prescription database, as none of the drugs are available over the counter. A limitation is, however, that we do not have data on whether the drugs are actually taken. Further, this study reveals no information on indications for the psychotropic drugs at an individual level, which would have been valuable complementary information in the discussion of the observed trends in use.

### Decline in use of alimemazine in young children – trend in use is in accordance with recommendations

Since 2004, use of alimemazine has almost halved in 0–17 year olds, attributed to a decrease among 1–2 year olds in particular, in whom use decreased more than threefold. Alimemazine is licensed and has long been used for childhood insomnia in Norway [15, 16, 22].

However, frequent use of alimemazine as a hypnotic in children has been questioned for several reasons [15]. Alimemazine is not licensed or recommended for use in children less than 2 years old because of indications of highly variable pharmacokinetics [24], heavy hangovers as a commonly reported side effect [25–27], and a few reports of fatal adverse reactions [28–32]. Although not commonly conducted in children, a few small randomized controlled trials have explored the hypnotic effect of alimemazine [33–36]. These showed that the effect was at most small, transient and associated with a “rebound” phenomenon on withdrawal. Thus, alimemazine is a much-debated drug, and a recently published paper concluded that the documented effects and safety of use as a hypnotic drug were inadequate to support use among children [15]. The information on lack of efficacy and negative side effects presented in both the media and the scientific literature as well as that reported directly to doctors may provide some explanation of the reduction in the use of alimemazine in children. Interestingly, decreased use of alimemazine among the youngest was not compensated by increasing use of alternative hypnotics/sedatives, or sedative antipsychotics, which remained stable in the youngest children in the period.

### Increasing use of hypnotic drugs in adolescents only

A previous study has documented increasing use of hypnotic drugs between 2004 and 2011 in 0–17 year olds in Norway, attributed to increasing use of melatonin [14]. The present study adds more detailed information to the phenomenon of increasing hypnotic drug use, as it reveals information on trends in use over time according to specific years of age. In summary, the increasing use of hypnotic/sedative drugs earlier reported in the total child and adolescent population is attributed to increasing use among adolescents only, and the oldest girls in particular. For example, their use among 17-year old girls increased threefold from 2004 to 2013, and they were used by 2-3 % of all 17-year old Norwegians in 2013. Thus, whereas use of alimemazine, the preferred sedative among the youngest, decreased, use of other hypnotics increased among adolescents. The observed increase in hypnotic drug use in adolescents may be explained by an increasing prevalence of sleeping problems. In a Norwegian repeated cross-sectional study in 1983 and 2005, prevalence of sleep-onset difficulties among adolescents increased during the last two decades [37]. As that study does not cover the time period of the present study, we cannot conclude that an increasing prevalence of sleeping problems is the explanation. An earlier study showed that melatonin was the main hypnotic drug used by adolescents in Norway [14]. A recently published study exploring morbidity in adolescent melatonin users revealed that 90 % of all long-term users had either a psychiatric or neurological diagnosis from the specialist health care system [38]. Thus, increasing use of melatonin in treating secondary sleep problems seems to be an important contributing factor in the observed increase in the overall use of hypnotic drugs in adolescents over time.

Pharmacoepidemiological studies on the use of hypnotic/sedative drugs in children and adolescents are scarce, but a similar trend with increasing incidences of use among adolescents, and frequent use of melatonin, was observed in an Icelandic study [4]. Information retrieved from the Danish and Swedish prescription databases reveals parallel trends in increasing melatonin use in the age group 5–19 years [39, 40], attributed to use in secondary sleep problems also in Denmark [41].

### Use of stimulants over a 10-year span – has the incline reached a threshold?

In several European countries and the US, increasing use of psychotropic drugs in children and adolescents can be explained by an increasing use of stimulants [2–4, 42]. Level of use seems to vary between countries, including the Nordic countries [9]. In 2007, 1-year prevalence of use in Iceland among 7–15 year olds was almost three times higher than in Norway, and the level of use was twice that of Denmark and Sweden [9].

There is a higher prevalence of stimulant use in boys compared to girls, which is in accordance with studies on prevalences of ADHD-disorders [43], and drug use [9]. Girls are more likely to be inattentive without being hyperactive or impulsive, compared with boys [44]. Girls are thus less likely to show obvious problems, which may contribute to a lower diagnosis rate among girls in childhood [45, 46].

Interestingly, a comprehensive review of ADHD prevalence studies concluded that, during the past three decades, prevalence estimates did not vary as a function of time [43]. However, we observed an increase in use over time, especially among girls, which may be explained by increasing awareness, access to treatment or changing clinical practices as suggested by the review [43].

### Increasing use of antidepressants among adolescents – girls in particular

In parallel to increasing use of hypnotic/sedative drugs and stimulants, this study documents an increasing use of antidepressants among adolescent girls in particular in the post FDA and EMA warning period.

For example, prevalence of use among 14–17 year old girls increased by 70 % from 2006 to 2013. Almost 3 % of all 17-year old girls had an antidepressant dispensed in 2013 and were 2.4 more likely to use antidepressants compared to their male counterparts. After puberty, the probability of being depressed is reported to be two to three times greater for girls than for boys [47, 48] which corresponds well with our findings of AD use in adolescents.

Parallel trends and level of AD use over time are observed in a Danish prescription database study on SSRIs; the increase in use over time is driven by use in adolescents, and among girls in particular [10]. Interestingly, an opposite trend has been observed in Iceland, where antidepressant use decreased among 0–17 year olds in the period 2004 to 2007. Levels of AD use have, however, been high in Iceland, and declined to 23 per 1000 in 2007 in the overall age-group [4]. Thus, despite trends of increasing antidepressant use in Norway and Denmark, levels of use in children and adolescents are low in comparison with Iceland – as 2 and 4 out of every thousand Norwegian boys and girls, respectively, in this age interval were dispensed antidepressants in 2013. Despite increasing use of ADs, levels of use in Europe seem low compared to Iceland and the US [4,10,49,50]. Back in 2000, use of antidepressants among 0–19 year olds in the US (16 per 1000) exceeded that of three Western European countries by at least threefold (1.1-5.4 per 1000) [50].

Several factors may explain increasing use. First, it may be attributed to an increasing prevalence of mental distress, and thus adolescents seeking help for their problems. In line with this, an increasing proportion of 15–16 year old adolescent participants in a Norwegian county survey reported seeking help for mental health problems [51]. Second, increasing AD use may be due to a more liberal prescription regime. In 2004–05, there were warnings against use of SSRIs in this population due to concerns about increased risk of suicidal ideation and behaviour [17, 18], and prescription of ADs among children and adolescents declined accordingly from 2004 to 2005 [52]. Since then, and according to a recently published systematic review from the Cochrane Collaboration, newer antidepressants may be beneficial in the treatment of depressive disorders in children and adolescents, although uncertainty still exists concerning risks and benefits [53]. From 2009, the SSRI fluoxetine was approved for treatment of depression in children > 8 years in Norway, and Norwegian guidelines for management of pediatric depression suggest that SSRIs may be indicated in moderate/severe depression and relapse of depression, always in conjunction with psychosocial interventions [54].

### Increasing use of antipsychotics – parallel trends to stimulant use?

Overall, this study revealed an overall relative increase in antipsychotic drug use of around 40 % in both genders from 2004 to 2013, attributed to increasing use from around the age of 8 and onwards. A parallel trend of increasing antipsychotic drugs in children and adolescents has been observed in Europe, the US, Canada, and Australia [3, 12, 13, 55–57]. One explanation for this could be an increasing use of antipsychotic drugs in ADHD or autism [12, 13, 55, 58].

### Anxiolytics for fever – cramps in young children

Anxiolytics were most frequently used by 1–2 year olds. Further analysis revealed that close to all users at this age had rectal solutions of benzodiazepines dispensed, commonly used to manage fever-cramps. Use of anxiolytic drugs in 0–17 year olds in Norway is somewhat higher than in Iceland and Denmark [4, 33].

## Conclusion

Different trends in psychotropic drug use exist in age and gender subgroups. Psychotropic drug use has decreased among the youngest children, attributed to alimemazine, and increased in older children and adolescents, attributed mainly to stimulants and hypnotics/sedatives. There is a higher level of psychotropic drug use in younger boys compared to girls, but there is a shift towards girls using more psychotropic drugs than boys during adolescence for all psychotropic drugs except stimulants.

## Abbreviations

FDA: Food and Drug Administration
EMA: European Medicines Agency
NorPD: Norwegian Prescription Database
ATC: Anatomical therapeutic chemical classification system of dugs
ADHD: Attention deficit and hyperactivity disorder
AD: Antidepressant drug
SSRI: Selective serotonin reuptake inhibitors
